# Serum concentration of ketamine and antinociceptive effects of ketamine and ketamine-lidocaine infusions in conscious dogs

**DOI:** 10.1186/s12917-016-0815-4

**Published:** 2016-09-09

**Authors:** Ubedullah Kaka, Bullo Saifullah, Adamu Abdul Abubakar, Yong Meng Goh, Sharida Fakurazi, Asmatullah Kaka, Atique Ahmed Behan, Mahdi Ebrahimi, Hui Cheng Chen

**Affiliations:** 1 Department of Veterinary Clinical Studies, Faculty of Veterinary Medicine, Universiti Putra Malaysia, 43400 Serdang, Selangor, Malaysia; 2 Department of Surgery and Obstetrics, Faculty of Animal Husbandry & Veterinary Sciences, Sindh Agriculture University Tandojam, Sindh, 70060 Pakistan; 3 Material Synthesis and characterization laboratory, Institute of Advanced technology, Universiti Putra Malaysia, 43400 Serdang, Selangor, Malaysia; 4 Department of Veterinary Preclinical Sciences, Faculty of Veterinary Medicine, Universiti Putra Malaysia, 43400 Serdang, Selangor, Malaysia; 5 Institutes of Tropical Agriculture, Universiti Putra Malaysia, 43400 Serdang, Selangor, Malaysia; 6 Laboratory of Vaccines and Immunotherapeutics, Institute of Bioscience, Universiti Putra Malaysia, 43400 Serdang, Selangor, Malaysia; 7 Department of Human Anatomy, Faculty of Medicine and Health Science, Universiti Putra Malaysia, 43400 Serdang, Selangor, Malaysia; 8 Department of Animal Sciences, Faculty of Agriculture, Universiti Putra Malaysia, 43400 Serdang, Selangor, Malaysia; 9 Faculty of Animal Husbandry & Veterinary Sciences, Sindh Agriculture University Tandojam, Sindh, 70060 Pakistan

**Keywords:** Ketamine, NMDA receptor, Nociceptive mechanical thresholds, Dog, Lidocaine

## Abstract

**Background:**

Central sensitization is a potential severe consequence of invasive surgical procedures. It results in postoperative and potentially chronic pain enhancement. It results in postoperative pain enhancement; clinically manifested as hyperalgesia and allodynia. N-methyl-D-aspartate (NMDA) receptor plays a crucial role in the mechanism of central sensitisation. Ketamine is most commonly used NMDA-antagonist in human and veterinary practice. However, the antinociceptive serum concentration of ketamine is not yet properly established in dogs. Six dogs were used in a crossover design, with one week washout period. Treatments consisted of: 1) 0.5 mg/kg ketamine followed by continuous rate infusion (CRI) of 30 μg/kg/min; 2) 0.5 mg/kg ketamine followed by CRI of 30 μg/kg/min and lidocaine (2 mg/kg followed by CRI of 100 μg/kg/min); and 3) 0.5 mg/kg ketamine followed by CRI of 50 μg/kg/min. The infusion was administered up to 120 min. Nociceptive thresholds and ketamine serum concentrations were measured before drug administration, and at 5, 10, 20, 40, 60, 90, 120, 140 and 160 min after the start of infusion.

**Results:**

Maximum concentration recorded was 435.34 ± 26.18 ng/mL, 582.34 ± 227.46 ng/mL and 733.77 ± 133.6 ng/mL for K30, KL30 and K50, respectively. The concentration at 120 min was 250.87 ± 39.87, 221.73 ± 91.03 and 343.67 ± 63.21 ng/mL at 120 min in K30, KL30 and K50, respectively. All the three infusion regimes maintained serum concentrations above 200 ng/mL. The thresholds returned towards baseline values within 20 min, after cessation of infusion.

**Conclusion:**

Serum concentration to produce mechanical antinociceptive effects in dogs is between 100 and 200 ng/mL. All the three infusion regimes in this study provided antinociceptive effects throughout the infusions. In this study, we found that the serum concentration of ketamine to produce mechanical antinociceptive effects in dogs is above 200 ng/mL. All three infusion regimes provided antinociceptive effects throughout the infusions without causing harmful effects. Further studies are recommended in a clinical setting.

## Background

The glutamate-activated *N-methyl-D-aspartate* (NMDA) receptor plays a crucial role in the development of central sensitization. Some of the characteristics associated with central sensitization are manifested clinically by a painful sensation from a non-painful stimulus (allodynia) and augmented pain sensation from previously painful stimuli (hyperalgesia) [1–5].

Association of the NMDA receptor with postoperative pain ushered a new chapter in the exploration of NMDA-antagonists for postoperative analgesia protocol. Ketamine is well established NMDA- antagonist. It is commonly used as an anaesthetic agent in children and animals. It is useful for its anti-nociceptive properties in acute pain, and can be used for chronic and neuropathic pain as well [4]. Ketamine has prevented hyperalgesia, improved analgesia [6–8] and decreased postoperative opioid consumption [8–10] in surgical patients.

The analgesic dose range of ketamine for use in dogs has not been properly established. As a consequence, analgesic benefits of ketamine are not yet fully recognized in dogs. The effective dose range of ketamine in human surgical patients has been established at a loading dose of 0.5 mg/kg, followed by continuous rate infusion (CRI) of 2 to10 μg/kg/min [7, 10–12]. In the absence of appropriate analgesic dose range, the doses used in studies of dogs [13–15] have been taken from human studies [16, 17]. This has resulted in reporting inconclusive results. Moreover, most of the studies were clinical. Consequently, the use of opioids pre and postoperatively in these studies might have masked the analgesic effects of ketamine. Apart from this, various scoring scales used in these studies have not been validated for reliability, specificity or linearity. Most importantly these scales were based on subjective assessment of behaviour [18, 19], which can be biased by inter-observer variability. Furthermore, none of these studies correlated effects with serum concentrations of ketamine.

So far, only one study reported plasma concentration versus antinociceptive actions in dogs [20]. In this study, ketamine was administered at 0.5 mg/kg, followed by 10 μg/kg/min for 59 min in dogs, antinociceptive effect was demonstrated only for the first four minutes, at which time the plasma concentration was >100 ng/mL. Plasma concentration of ketamine declined to <100 ng/mL after 5 min [20]. Further analysis revealed that the plasma concentrations of ketamine at pseudo-steady state were five times lower than the expected concentrations to that in humans receiving the same CRI. They concluded that this dose was insufficient to maintain the analgesic concentration of the ketamine. This also suggests that ketamine pharmacokinetics in dogs may differ from that in humans, and that may warrant the use of higher infusion rates in dogs.

Lidocaine is commonly used as a local anaesthetic and anti-arrhythmic agent in veterinary and human medicine. With the mechanism based approach combination of lidocaine acting on sodium channels, which play a part in generation and transmission of nociceptive impulses [1] and ketamine acting on NMDA receptors would be better strategy to manage postoperative pain, compared to conventional method of administering single drug for post operative pain management. The major benefit of combining lidocaine and ketamine is to prevent the development of central sensitization during surgical intervention [21], which may reduce pain and discomfort in the postoperative period. The combination of ketamine and lidocaine has been reported to decrease the minimum alveolar concentration (MAC) of inhalant anaesthetics in dogs [22–25]. It has been suggested that the MAC reducing effect may be due to analgesic or sedative effect or both [22, 24, 25]. The analgesic effects of the combination of lidocaine and ketamine in conscious dogs have not been reported.

This study therefore, aimed to correlate the serum concentrations of ketamine to the antinociceptive effects using two infusion rates of ketamine. Furthermore, this study also evaluated the clinical and antinociceptive effects of incorporation of lidocaine infusion to ketamine in a controlled, repeatable situation.

## Methods

### Animals and treatments

Six adult healthy mix-breed female dogs weighing 17.2 ± 2.2 kg (Mean ± SD) with the age of 2 to 10 years old were used in this study. Animals were obtained from a local stray animal holding facility following informed consent. These animals were acclimatized for one month, during this period they were kept under observation for any disease or abnormality. Dogs were judged healthy based on physical examination, hematology, and blood biochemistry, and have not been showing any sign of illness during one month of previous observation. Animals were brought daily to the experimental room, which was adjacent to the kennels, in order to familiarise them with the experimental station. Experiments were conducted in animal experimental station in a quiet environment with minimum number of observers. Experiments were conducted in day time, starting at 8:00 am. The study was approved by the Universiti Putra Malaysia Animal Care and Utility Committee (Reference Number UPM/IACUC/AUP-R023/2013).

All six dogs were subjected to three treatment protocols in a randomized crossover design with one-week washout period between the treatments. Treatment protocols were: 1) 0.5 mg/kg ketamine (NARKETAN®-10, 100 mg/mL, Vetoquinol UK Limited, Buckingham, UK) followed by continuous rate infusion (CRI) of 30 μg/kg/min (K30); 2) Combination of ketamine (0.5 mg/kg followed by CRI of 30 μg/kg/min) and lidocaine hydrochloride (Xylocaine® 2 % AstraZeneca, France) 2 mg/kg followed by CRI of 100 μg/kg/min (KL30); and 3) 0.5 mg/kg ketamine followed by CRI of 50 μg/kg/min (K50).

The loading doses of K30, KL30 and K50 were mixed in normal saline to a final volume of 3 mL and injected over one minute. Ketamine was diluted with normal saline to 5 mg/mL and lidocaine 16.66 mg/mL for infusion. Infusions were delivered for 120 min through a precision syringe pump (Omnifuse, Graseby medical limited, United Kingdom). Right and left cephalic veins were catheterized with 20 G indwelling catheters (Vasofix® Braunule®, B. Braune Melsungen AG 34209 Melsungen, Germany) for blood sampling and infusion of drugs, respectively. Prior to the actual study, the infusion regimens were tested in two of the six dogs.

### Algometer device

Wagner algometer (FPX 25, Wagner Instruments, Greenwich CT, USA) with a modified tip was used in this study.

### Nociceptive mechanical thresholds

The nociceptive mechanical thresholds were recorded in duplicates from the carpal pad, metacarpal footpad, tibia and femur of right limbs, and the abdomen. Thresholds were determined before treatment as baseline and then after start of treatment at 10, 20, 40, 60, 90, 120, 140 and 160 min. These body points were selected based on models used in the previous studies [26–28] conducted in dogs. The carpal pad and metacarpal pad were tested by applying the tip of the algometer at midpoint of these pads. The tibia was tested on the distal latero-dorsal surface where bone could be palpated through the skin. The femur was tested on the distal latero-dorsal surface, where the bone could be palpated through the skin by displacing the muscle with little pressure applied with fingers. The abdomen was tested on the midline at the midpoint between umbilicus and pubis. The order of testing was firstly the metacarpal pad, followed by carpal pad, femur, tibia, and finally, the abdomen. At each testing point, the tip was placed perpendicular to the surface area. The pressure was increased gradually until the dog responded. The entire threshold recording was performed by the same researcher. During thresholds determination, the operator did not look at the reading of the algometer as he applied consistent force. Instead, he concentrated on the dogs’ response and immediately stopped at response. The end-points were cross-checked by another observer who took note of both the algometer readings and dogs’ response. The most common response identified during the preliminary trial was clear withdrawal reflex of the limb, vocalization, withdrawal of the limb accompanied with vocalization and guarding of the abdomen. According to Le Bars et al. (2001), in order to avoid tissue damage, cut-off pressure should be set at three times the thresholds of control [29]. Preliminary trials and the baseline thresholds on body points tested in this study were 5 to 6 Newtons (N). Thus, the cut-off pressure was set at 18 N for this study. If no reaction was perceived before the cut-off limit, this value (18 N) was recorded as the mechanical nociceptive threshold. One of the dogs did not respond to application of the algometer up to the cut-off point of 18 N at its abdomen, thus, was not tested at abdomen for all treatment groups.

### Sedation score and side effects

A composite sedation score and a behavioural chart adopted from Bergadano et al. (2009) were used to evaluate the psychomimetic side effects of ketamine at the measurement time points [20]. The sedation score on a 0 (no sedation) to 12 (deep sedation) scale, was assigned by adding the ranking of different descriptors (Table 1). The incidence of psychomimetic signs (disorientation, lateral head movements, fixed eyes, apneustic respiration, salivation, tremors and hyperactivity) was noted for each treatment group. The person involved in scoring sedation and side effects was blinded to the treatments.

**Table 1:** Sedation score scale used in this system (Adopted from Bergadano et al. 2009)

Score	Consciousness	Eye	Responsiveness	Relaxation
0	Awake	Not rotated	Respond to voice	Moves spontaneously
1	Aware	Moderate rotation	Respond to gentle touch	Relaxed, no shivering
2	Not aware but arousable	Rotated	Does not respond to touch	Very Relaxed
3	Not aware and not arousable	Nystagmus	Hyperexcitable	Hypertonous

### Blood sampling

Blood samples were taken before administration of treatment as baseline, and then at 1, 5, 10, 20, 40, 60, 90, 120 and 140 min after the start of infusion. At each time points, 2 mL of blood was sampled and kept in plain sampling tubes (BD Vacutainer®, BD Franklin Lakes NJ USA), and immediately placed on ice. Samples were centrifuged and serum was harvested, and then frozen at - 80 °C until analysis.

### High performance liquid chromatography analysis

Ketamine concentration in serum was determined using high performance liquid chromatography (HPLC). In brief, the system consisted of Waters HPLC 2695 separation module, an Agilent C18 column (5 μm particle size; 4.6 × 150 mm), photodiode array (PDA) detector and a computer equipped with Empower software (Waters, Milford, MA, USA).

### Extraction of drug from serum

The ketamine was extracted from serum by the method reported earlier (Doherty et al. 2007). Briefly, the frozen serum samples were thawed followed by vortex mixing and transferred to 1 mL of glass test tube containing 25 μL of (internal standard, 50 μg/mL) trimethoprim. Further, 200 μL of 1 molar solution (1 M) of sodium hydroxide (NaOH) and 5 mL methylene chloride (CH_2_Cl_2)_ were added to above samples. Tubes were vortex mixed again and then centrifuged for 20 min at 1500 g. The organic layer was separated and placed in clean tube evaporated with gentle stream of nitrogen till dryness. Finally, samples were reconstituted with 1 mL of mobile phase and 100 μL was injected for HPLC analysis.

### Sample analysis

In brief, freshly prepared isocratic mixture of 0.02 M potassium dihydrogen phosphate (KH_2_PO_4_) pH 4.5, adjusted with phosphoric acid and acetonitrile (ACN) (ACN:KH_2_PO_4_ (40:60) was used as mobile phase with flow rate of 1.0 mL/min. Calibration curves for serum analysis were prepared by spiking the dog serum with ketamine. A linear concentration range of 25–1500 ng/mL was established with r^2^ value of 0.99.

### Statistical analysis

Data are presented as mean ± standard deviation. Statistical analysis was performed using the SAS software package, version 9.3 (SAS Institute Inc., Cary, NC, USA). Prior to the analysis, data were checked for their conformance to the normal distribution using Kolmogrov-Simirnov test. Changes in mechanical thresholds and serum concentrations over time and across treatment groups were compared using the repeated-measures ANOVA model. Bonferroni adjusted *P* values were used when indicated by a significant *F* test (*P* < 0.05). Correlation between the mechanical thresholds and serum concentrations of ketamine was calculated with Pearson test. Effects of treatment over time on sedation score were analyzed using Friedman’s test. Overall significance was set at a value of *P* < 0.05. The observed power for the current study was determined to be 80.53 % for 1-Way ANOVA, and 90.75 % for Repeated Measure ANOVA. This was based on an effect size value of 0.8165, obtained by calculating the serum ketamine concentrations 1 min after administration (peak value), and at 120 min after administration. Power calculations were performed using G*Power Software, version 3.1.9.2 [30].

## Results

The experiments were well tolerated by all the dogs. Changes in behavior due to recognized psychomimetic effects of ketamine were mild and harmless. The behavioral changes disappeared within 20 min after the end of infusion, and the dogs resumed their activity immediately after the end of experiment. The algometer device was well tolerated by all the animals. Transient dimples were observed at the carpal pad, metacarpal footpad, and abdomen, which disappeared within 1 min, before the replicate measurement. There was no sign of redness, swelling, bleeding, exudates, and lameness during, immediately after, and 24 h after the experiment.

### Serum concentrations of ketamine

**Fig. 1:**
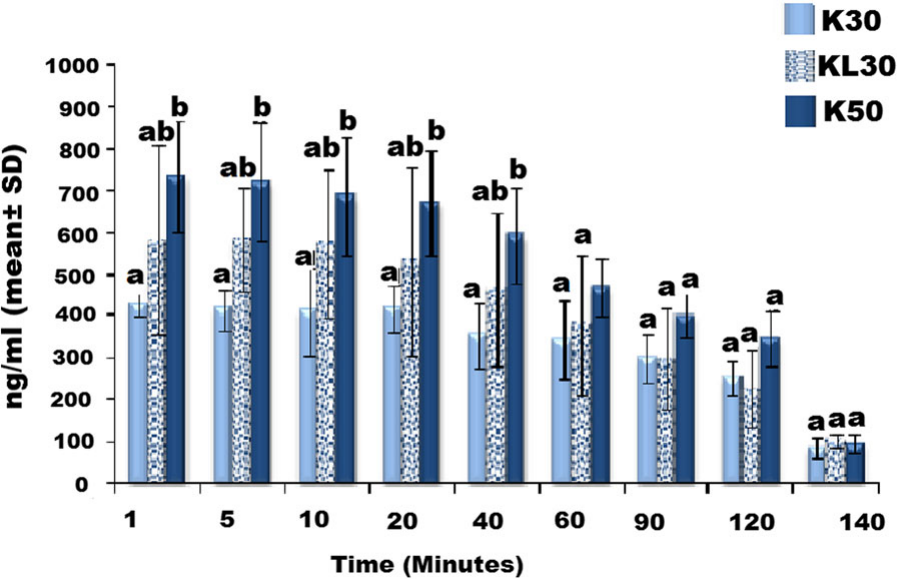
Comparison (Mean ± SD) between serum concentration of K30, KL30 and K50 in 6 dogs during and after CRI. K30 = Ketamine 0.5 mg/kg loading dose followed by 30 μg/kg/min, KL30 = Ketamine 0.5 mg/kg loading dose followed by 30 μg/kg/min and lidocaine 2 mg/kg loading dose followed by 100 μg/kg/min, K50 = Ketamine 0.5 mg/kg loading dose followed by 50 μg/kg/min CRI. At each time point, groups with similar alphabet are not different (*P* < 0.05)

The serum concentrations (Mean ± SD) of K30, KL30 and K50 across time points are illustrated in Fig. 1. Maximum concentration was observed at 1 min for K30 (435.34 ± 26.18 ng/mL), KL30 (582.34 ± 227.46 ng/mL), and K50 (733.77 ± 133.6 ng/mL), which remained stable for 20 min, and declined gradually reaching around 250.87 ± 39.87, 221.73 ± 91.03 and 343.67 ± 63.21 ng/mL at 120^th^ min in K30, KL30 and K50, respectively. The serum concentration decreased rapidly to less than 100 ng/mL within 20 min following the end of infusions in all three treatments. Between 1 and 40 min, serum concentration of K50 was higher than K30, while concentration of KL30 lied in between K50 and K30. By 60﻿﻿^th﻿﻿^ min, the three groups were not statistically different.

### Nociceptive mechanical thresholds

**Fig. 2:**
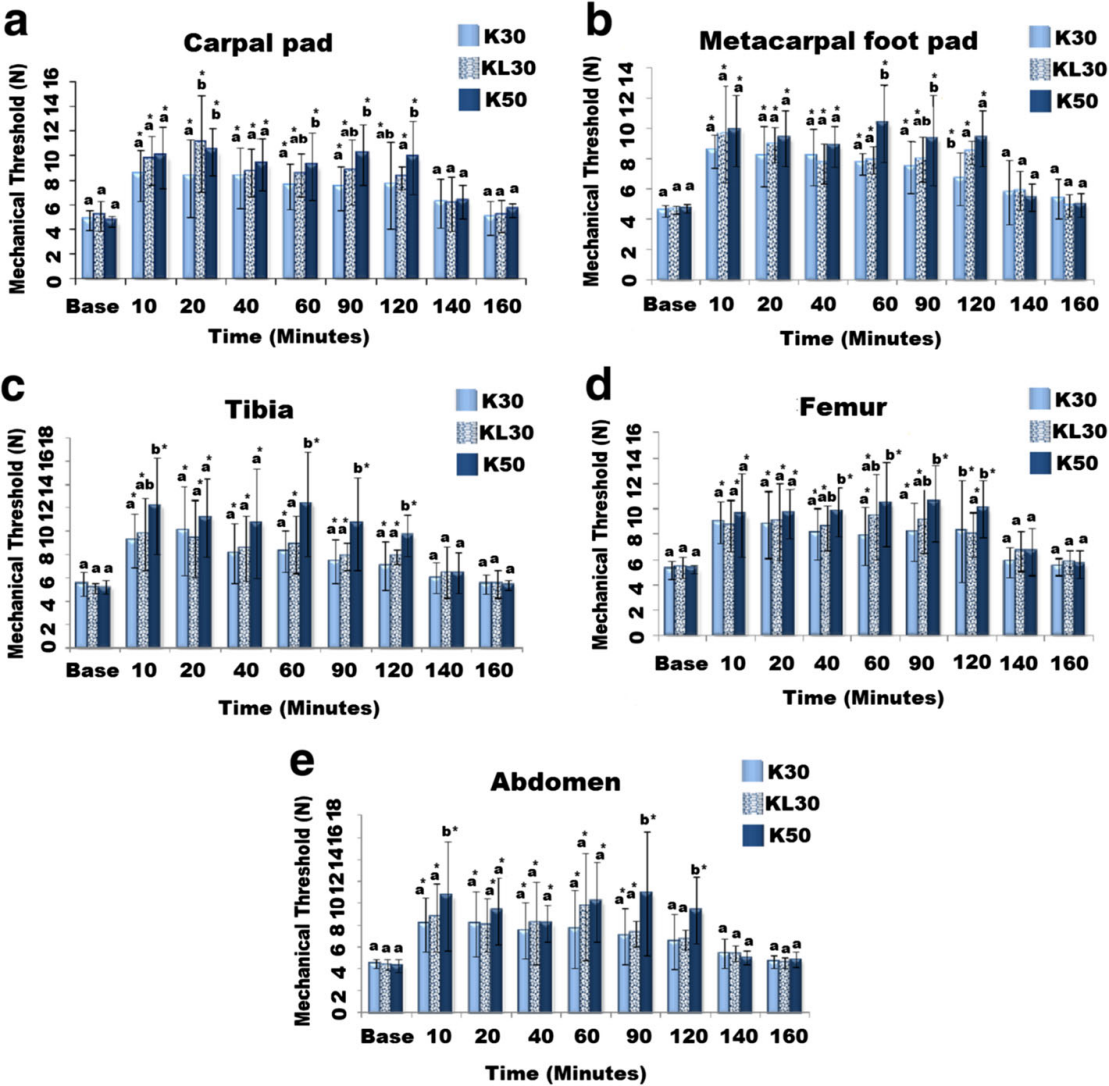
Effects of K30, KL30 and K50 on mechanical nociceptive thresholds measured at the carpal pad (**a**), metacarpal footpad (**b**), tibia (**c**), femur (**d**) (*n* = 6) and abdomen (**e**) (*n* = 5). K30 = Ketamine 0.5 mg/kg loading dose followed by 30 μg/kg/min CRI, KL30 = Ketamine 0.5 mg/kg loading dose followed by 30 μg/kg/min and lidocaine hydrochloride 2 mg/kg loading dose followed by 100 μg/kg/min CRI, K50 = Ketamine﻿ 0.5 mg/kg loading dose followed by 50 μg/kg/min CRI,. Data are expressed as mean ± SD. * denotes significant difference from baseline within treatment, at each time point, groups with similar alphabet are not different (*P* < 0.05)

There was no significant difference among the baseline mechanical thresholds at carpal pad, metacarpal foot pad, tibia, femur and abdomen in all treatment groups (Fig. 2a-e). All the three treatments increased thresholds significantly (*P* < 0.05) throughout the infusion up to 120 min. Mechanical thresholds returned to baseline within 20 min of the end of CRI at 140^﻿th﻿^ min. Overall, nociceptive mechanical thresholds were higher in KL30 than in K30 and also higher in K50 than in KL30, and some significant differences between groups were recorded. There was weak positive correlation between serum concentration of K30 with mechanical thresholds at carpal pad (r-0.24 *P* = 0.141), metacarpal footpad (r-0.32, *P* = 0.053), tibia (r-0.33, *P* = 0.047), femur (r-0.30, *P* = 0.075) and abdomen (r-0.35, *P* = 0.053). Likewise, weak positive correlation of K50 with mechanical thresholds at carpal pad (r-0.29 *P* = 0.080), metacarpal footpad (r-0.38, *P* = 0.020), tibia (r-0.18, *P* = 0.330), femur (r-0.22, *P* = 0.185) and abdomen (r-0.12, *P* = 0.527) were observed.

### Heart rate, body temperature and respiration

Baseline heart rates were not different amongst all treatments. Heart rates increased significantly from baselines in all treatments during the 120 min of treatment infusions. Following cessation of infusions, heart rates decreased, but remained higher than baselines in KL30 and K50. Heart rates between K30 and KL30 were not different, while K50 resulted in higher heart rates compared to K30 at 40 to 60 min and KL30 at 40 to 120 min (Table 2). Body temperatures increased significantly from baselines throughout the 160-minute study period in K30 and KL30 groups, and up to 120 min in K50 (Table 3). This increase in body temperature was within the normal range. The change in respiration was insignificant in K30, while KL30 and K50 resulted in increased respiratory rates at 120 to 160, and 20 to 160 min, respectively. Respiratory rate increased from 33 ± 7 at baseline, to as much as 93 ± 5 breaths per minute in KL30, and from 29 ± 7 at baseline, to as much as 93 ± 5 breaths per minute in K50 during infusion of treatment.

**Table 2:** Effects of K30, KL30 and K50 on heart rates (Mean ± SD) measured in 6 dogs using crossover design

	K30	KL30	K50
Baseline	79 ± 6	77 ± 7	81 ± 6
20 min	113 ± 24^a^	101 ± 19^a^	113 ± 22^a^
40 min	104 ± 16^a^	99 ± 18^a^	123 ± 19^abc^
60 min	105 ± 15^a^	104 ± 260^a^	124 ± 23^abc^
90 min	113 ± 15^a^	107 ± 21^a^	127 ± 25^ac^
120 min	106 ± 19^a^	98 ± 20^a^	114 ± 14^ac^
140 min	93 ± 20	93 ± 12^a^	99 ± 15^a^
160 min	86 ± 14	94 ± 11^a^	102 ± 25^ab^

*K30* Ketamine 0.5 mg/kg loading dose followed by 30 μg/kg/min CRI, *KL30* Ketamine 0.5 mg/kg loading dose followed by 30 μg/kg/min and lidocaine hydrochloride 2 mg/kg loading dose followed by 100 μg/kg/min CRI, *K50* 0.5 mg/kg loading dose followed by 50 μg/kg/min CRI. ^a^denotes significant difference from baseline within same treatment, ^b^denotes significant difference from K30 at corresponding values, ^c^denotes significantly different from KL30 at corresponding time points. Overall significance was set at a value of *P* < 0.05

**Table 3:** Effects of K30, KL30 and K50 on body temperatures (Mean ± SD) measured in 6 dogs using crossover design

	K30	KL30	K50
Baseline	38.6 ± 0.4	38.7 ± 0.3	38.8 ± 0.2
20 min	39.0 ± 0.5^a^	39.1 ± 0.6^a^	39.2 ± 0.6^a^
40 min	39.1 ± 0.6^a^	39.0 ± 0.5^a^	39.3 ± 0.5^a^
60 min	39.2 ± 0.4^a^	39.1 ± 0.4^a^	39.3 ± 0.5^a^
90 min	39.2 ± 0.4^a^	39.1 ± 0.4^a^	39.0 ± 0.4^a^
120 min	39.1 ± 0.5^a^	39.1 ± 0.5^a^	39.0 ± 0.3^a^
140 min	39.3 ± 0.5^a^	39.2 ± 0.4^a^	39.0 ± 0.3
160 min	39.0 ± 0.4^a^	39.1 ± 0.5^a^	39.0 ± 0.3

*K30* Ketamine 0.5 mg/kg loading dose followed by 30 μg/kg/min CRI, *KL30* Ketamine 0.5 mg/kg loading dose followed by 30 μg/kg/min and lidocaine hydrochloride 2 mg/kg loading dose followed by 100 μg/kg/min CRI, *K50* 0.5 mg/kg loading dose followed by 50 μg/kg/min CRI ^a^denotes significant difference from baseline within same treatment. Overall significance was set at a value of *P* < 0.05

### Sedation and side effects

Sedation score and side effects are presented in Tables 4 and 5. There was significant difference in the sedation score between K30, KL30 and K50 groups. The median sedation scores were 0 throughout the 160-minute study period in K30 and KL30. In contrast, median score in K50 were 3 between 1 and 20 min, and reduced to 1 between 40 and 90 min. Most of the dogs exhibited one or more side effects such as disorientation, swaying of the head, salivation and hyperactivity. These effects were observed throughout the duration of 120 min infusion and disappeared at 140^th^ min, which is within 20 min after the end of infusions. The side effects were less severe in K30 and KL30 compared to K50.

**Table 4:** Sedation score in median (IQR) values across each time points of the each group after administration of K30, K50, and KL30 in 6 dogs

Time (minutes)	K30	KL30	K50^a^
0	0 (0–0)	0 (0–0)	0 (0–0)
01	0 (0–0)	0 (0–0)	3 (0–3)
05	0 (0–0)	0 (0–0)	3 (0–3)
20	0 (0–0)	0 (0–0)	3 (0–3)
40	0 (0–0)	0 (0–0)	1 (0–3)
60	0 (0–0)	0 (0–0)	1 (0–3)
90	0 (0–0)	0 (0–0)	1 (0–3)
120	0 (0–0)	0 (0–0)	0 (0–0)
140	0 (0–0)	0 (0–0)	0 (0–0)
160	0 (0–0)	0 (0–0)	0 (0–0)

*K30* ketamine 0.5 mg/kg loading dose followed by 30 μg/kg/min, *K50* 0.5 mg/kg loading dose followed by 50 μg/kg/min, *KL* = ketamine 0.5 loading dose followed by 30 μg/kg/min + lidocaine 2 mg/kg loading dose followed by 100 μg/kg/min. Overall significance was set at a value of *P* < 0.05. ^a^K50 is significantly different from K30 and KL at *P* < 0.003 (Friedman test)

**Table 5:** Frequency of side effects (number of dogs out of six showing a specific psychomimetic sign)

		0 min	1 min	5 min	20 min	40 min	60 min	90 min	120 min	140 min	160 min
Disorientation/confused	K30	0	5	5	4	3	3	3	3	0	0
	K50	0	5	5	6	6	6	5	6	0	0
	KL30	0	4	5	5	5	5	5	5	0	0
Lateral head movements	K30	0	3	3	2	2	2	2	2	0	0
	K50	0	5	5	6	6	6	5	5	0	0
	KL30	0	3	4	4	4	4	4	4	0	0
Glazy Eyes	K30	0	0	0	0	0	0	0	0	0	0
	K50	0	0	0	0	0	0	0	0	0	0
	KL30	0	0	0	0	0	0	0	0	0	0
Apneustic respiration	K30	0	0	0	0	0	0	0	0	0	0
	K50	0	0	0	0	0	0	0	0	0	0
	KL30	0	0	0	0	0	0	0	0	0	0
Salivation	K30	0	4	4	4	4	4	4	4	0	0
	K50	0	4	4	4	4	4	4	4	0	0
	KL30	0	4	4	4	4	4	4	4	4	0
Tremors	K30	0	0	0	0	0	0	0	0	0	0
	K50	0	0	0	0	0	0	0	0	0	0
	KL30	0	0	0	0	0	0	0	0	0	0
Hyperactivity	K30	0	1	1	1	1	1	1	1	0	0
	K50	0	3	3	3	3	3	3	3	0	0
	KL30	0	2	2	2	1	1	0	0	0	0

K30 ketamine 0.5 mg/kg loading dose followed by 30 μg/kg/min, K50 0.5 mg/kg loading dose followed by 50 μg/kg/min, KL30 ketamine 0.5 loading dose followed by 30 μg/kg/min + lidocaine 2 mg/kg loading dose followed by 100 μg/kg/min

## Discussion

The concentrations of ketamine in the present study were 435.34 ± 26.18 ng/mL for K30, 582.34 ± 227.46 ng/mL for KL30 and 733.77 ± 133.6 ng/mL for K50 at 1 min and 250.87 ± 39.87, 221.73 ± 91.03 and 343.67 ± 63.21 ng/mL at 120 min for K30, KL30 and K50, respectively. The serum concentrations of ketamine produced by all the 3 CRI regimens in this study could be maintained above 100 ng/mL during the 120- min infusions. Furthermore, nociceptive thresholds at most body points were raised above baseline values during the 120 min of infusions. The nociceptive thresholds returned towards baseline values within 20 min after cessation of infusions, at which point, serum levels were found to be lower than 100 ng/mL. Results from this study suggested that the minimum concentration of ketamine to produce analgesia in dogs is between 100 as observed at 140 min and 200 ng/mL during infusion. This concurred with findings in humans, where serum concentration of 100–200 ng/mL of ketamine was reported to be analgesic with minimal side effects [11, 31].

Effective analgesic concentration of ketamine has not been reported in dogs. The only study reporting the analgesic concentration of ketamine versus its antinociceptive action in dogs has been conducted by Bergadano et al. [20]. They found that the analgesic effect only lasted for the first four minutes at plasma concentration of 220–370 ng/ mL. No further analgesic effect was observed thereafter for up to 59 min, when plasma concentration was found to be below 100 ng/mL. They selected a ketamine dose of 0.5 mg/kg and 10 μg/kg/min from the human studies [16, 17, 31, 32]. However, these dosages had difficulties to sustain a desired plasma ketamine concentration (i.e. above 100–200 ng/mL) to produce antinociceptive effect conscious dogs. The authors suggested that this variation was due the difference in pharmacokinetics of ketamine between human and dogs, in addition to inter-species an intra species difference has also been reported in dogs [33]. Various factors could contribute to the individual variations within the species; these include age, body weight, lean-to-fat body ratio and genetics [34–36]. Although there is no data reporting the influence of genetic factors on the disposition of ketamine in dogs, its potential role cannot be excluded. In this study, we selected the ketamine dose based on the prior results reported by Bergadano et al. [20] . We used similar loading dose of 0.5 mg/kg, but increased the CRI to 30 and 50 μg/kg/min, doses 3 and 5 times higher than that used by Bergadano. Both the doses maintained serum concentration above the level required to raise the mechanical nociceptive thresholds up to 120 min. Our study showed that the mechanical nociceptive threshold in dogs can be raised through ketamine infusion. In order to maintain this plasma concentration and have an antinociceptive effect over 120 min, higher infusions rates than previously employed by Bergadano et al. in dogs (20) are deemed necessary. A time of 120 min was selected in our study to simulate a multimodal analgesic approach that could provide pre-intra and post-operative antinociception for a potential surgery of 45 min. This period covers the time before, during and after surgery up to recovery. Thus, results of this study confirmed the need for higher infusion rates of ketamine in dogs than that previously employed by Bergadano in dogs [20].

In the present study, the concentration of ketamine was higher during first 10–20 min of adminsitration, and declined gradually thereafter. The initial hike and maintenance of the higher serum concentrations were likely contributed by the loading dose. Since ketamine is a high clearance drug, it requires continuous infusion for at least five half lives, which is approximately 10 h to maintain steady state [37]. Ketamine also increases sympathetic nervous system activity, resulting in increased cardiovascular functions and hepatic blood flow [38, 39]. Thus, the elevated heart rates in the present study are likely a reflection of sympathetic stimulation by ketamine.

Nociceptive mechanical thresholds increased significantly compared to baseline after administration of the K30, KL30 and K50 groups and remained elevated throughout the 120-minutes of infusion. The thresholds returned to baseline at 140 min, that is, within 20 min after the end of infusion. Mechanical thresholds have been used in previous studies in animals [14, 26–28, 40–45] for the objective quantification of pain and to evaluate the analgesic efficacy of drugs. The analgesic effect of the test drug reported by these studies was either increase in the mechanical thresholds at the site of test compared to baseline [26, 28] or concurrent rise in the mechanical thresholds equal to that produced by positive control analgesic or dose used to compare with [40, 45] or equal rise in mechanical thresholds at the site of surgery by the same drug compared for two routes of administration [40, 44]. Kukanich et al. (2005) and Kaka et al. (2015) reported increase in mechanical thresholds after administration of intravenous morphine at 1 mg/kg [26, 28]. The increase in mechanical thresholds compared to baseline in these studies was suggestive of analgesic action of morphine. In the present study mechanical thresholds increased significantly compared to baseline in K30, KL30 and K50 treatments throughout the infusion period of 120 min and returned to baseline within 20 min after the end of infusion. The rise in mechanical thresholds suggests the antinociceptive actions of K30, KL30 and K50 throughout the duration of infusion. The modified algometer and technique used in this study have been validated previously in our lab [28]. In this study, conscious vocalization may not be a good indicator of nociceptive threshold as it occurred only in 2 of subjects, and therefore we depended on algometer readings, even though vocalization has been accepted as part of the pain study protocol in human beings.

Mechanical pressure for quantification of surgical pain or hyperalgesia is termed as “algometry”. Algometry has been used to identify abnormal pain thresholds and to monitor response to analgesic treatment in animals and humans [46–49]. Algometry has also been used to evaluate the analgesic effects of drugs in dogs [26, 27, 40]. Rate of force of application plays a crucial part on the reliability of this method. Application of force at a faster rate may provoke a low false threshold reading [50]. In a study of reliability and validity of algometer Kinser et al. (2009) concluded that this is highly reliable and valid method [51]. The authors suggested that with some practice, an individual becomes reliable in applying force with an algometer. In this study researcher who operated the algometer has sufficient practice in applying the rate of force of application during preliminary experiments, which have been demonstrated as consistent results in saline treatment during validation of the method [28]. Similarly, in this study no significant difference in mechanical thresholds between control and 140 min indicates the reliability of results. Thus, the results of the above studies suggest that algometry is reliable and valid technique for the quantification of mechanical nociceptive thresholds. It should be noted that despite an open label approach of the current study, potential operator bias was minimized by blinding the operator of the algometer to the threshold values. Only another nearby observer who was not operating the algometer was entrusted to record the readings displayed on the LCD screen. It should be noted that the operator of the algometer, cannot see the values projected on the LCD screen. We are aware that open label studies have the limitation that results could be refined and biased [51], however, we tried to minimise the bias by blinding to the treatment the investigator who evaluated sedation and side effects of the infusions, and the investigator operating the algometer to the values recorded.

Ketamine has been reported to increase body temperature in dogs [23]. Effects of body temperature on mechanical thresholds in dogs have not been reported. In this study body temperatures increased significantly from baseline throughout study, however, this increase was within the normal range. The increase in mechanical thresholds throughout infusion and decrease in the mechanical thresholds after the end of infusion, at which time the temperature was still higher than baseline suggests that increase in temperatures did not affect the mechanical thresholds.

Ketamine is well known for its psychomimetic effects in dogs; these effects range from hypersalivation to excitability and delirium. The dose regimens used in this study did produce such side effects; however they were harmless and ceased within 20 min of ending the infusions. Furthermore, side effects were less intense and occurred less frequently in K30 and KL30 compared to K50. The sedation score recorded in this study was as low as 0 in K30 and KL30, and as high as 3 in K50, which is mild and comparable to the scores reported by Bergadano et al. [20].

Commonly used potent analgesics reduce the minimum alveolar concentration (MAC) of inhalant anaesthetics in dogs and humans [52, 53] ; the reduction in MAC of inhalant anaesthetic has been suggested either due to analgesic or sedative effects of the drug. Hence, if a drug decreases the MAC, it should be tested for its analgesic effects in awake patients [54]. The dosage of lidocaine selected in this study was based on MAC studies in dogs under anaesthesia [25] and studies in conscious dogs [55]. In the study on conscious dogs, lidocaine CRI of 100 μg/kg/min up to four hours did not produce any side effect [55]. Therefore, lidocaine at 2 mg/kg loading dose and CRI 100 μg/kg/min was combined to ketamine in this study. This combination of lidocaine and ketamine in present study was tested in preliminary study on two dogs before actual experiments. The major benefit of combining lidocaine and ketamine is to decrease the drug related side effects [56], decrease opioid requirement [57] intensity of pain in postoperative period due to central sensitization during surgical intervention [21]. The addition of lidocaine at 2 mg/kg loading dose and CRI of 100 μg/kg/min to ketamine infusion in this study resulted in higher mean nociceptive threshold values only at carpal pad and metacarpal footpad. Whereas, no difference in the serum concentration between K30 and KL 30 as well as KL 30 and K50 was observed due to addition of lidocaine to ketamine. The inherent high individual variability in these parameters would require large sample size to demonstrate statistical difference, which was not feasible in this study.

In this study, overall nociceptive thresholds were relatively higher in K50 than K30. Suggesting that K50 provided slightly more analgesia than K30. On the other hand, there was no such difference between KL30 and K50. Thus, K 50 and KL30 demonstrated more or less similar effect on nociceptive thresholds than K30. Nevertheless, all the three regimens significantly raised nociceptive thresholds from baseline thresholds, suggesting significant analgesic effects demonstrated by the three regimens used. Further studies are required to investigate the analgesic effects of these three doses in dogs undergoing surgical procedures.

## Conclusions

All the three treatment regimes in this study maintained serum concentrations of ketamine above 200 ng/mL and raised mechanical nociceptive thresholds at most body points during the 120 min of infusions. A linear correlation between the mechanical thresholds and serum concentrations of ketamine was found. Results from this study suggested that the minimum concentration of ketamine to produce analgesia in dogs is between 100 and 200 ng/mL. All the three treatments provided antinociceptive effects throughout the infusions. In this setting the addition of lidocaine to ketamine increased mechanical thresholds without any further side effect. Clinical studies are encouraged to evaluate the analgesic effects of ketamine CRI and combination of lidocaine and ketamine at these dose regimens.
